# AURKA upregulation plays a role in fibroblast-reduced gefitinib sensitivity in the NSCLC cell line HCC827

**DOI:** 10.3892/or.2015.3764

**Published:** 2015-01-29

**Authors:** JIA CHEN, HUIQI LU, WANG ZHOU, HUABIN YIN, LISHUANG ZHU, CHANG LIU, PENGFEI ZHANG, HUIMIN HU, YILI YANG, HUANXING HAN

**Affiliations:** 1 Translational Medicine Center, Changzheng Hospital, Affiliated to The Second Military Medical University, Shanghai, P.R. China; 2 Department of Bone Tumor Surgery, Changzheng Hospital, Second Military Medical University, Shanghai, P.R. China; 3 Cancer and Developmental Biology Laboratory, National Cancer Institute, Frederick, MD, USA

**Keywords:** fibroblasts, Aurora-A kinase, gefitinib, resistance, NSCLC

## Abstract

Epidermal growth factor receptor (EGFR) tyrosine kinase inhibitors (EGFR-TKIs) have been used to treat non-small cell lung carcinoma (NSCLC) patients that have EGFR-activating mutations. EGFR-TKI monotherapy in most NSCLC patients with EGFR mutations who initially respond to EGFR-TKIs results in the development of acquired resistance. We investigated the role of fibroblasts in stromal cell-mediated resistance to gefitinib-induced apoptosis in EGFR-mutant NSCLC cells. While gefitinib induced apoptosis in EGFR-mutant NSCLC cells, apoptosis induction was diminished under stromal co-culture conditions. Protection appeared to be mediated in part by Aurora-A kinase (AURKA) upregulation. The protective effect of stromal cells was significantly reduced by pre-exposure to AURKA-shRNA. We suggest that combinations of AURKA antagonists and EGFR inhibitors may be effective in clinical trials targeting mutant EGFR NSCLCs.

## Introduction

Lung cancer is the leading cause of cancer-related death worldwide with non-small cell lung carcinoma (NSCLC) accounting for ~80% of lung cancers (1). Activating mutations of the epidermal growth factor receptor (EGFR) occur in 30–40% of the patients with NSCLC in China, and are associated with poor prognosis (2). EGFR mutations result in constitutive activation of the EGFR in the absence of the EGF ligand and abnormal activation of downstream signaling pathways, including mitogen-activated protein kinase (Mek)/extracellular signal regulated kinase (ERK) and phosphatidylinositol-3 kinase (PI3K) (3–5). Activation of these downstream effectors upregulates Mcl-1, Bcl-XL and survivin, allowing cancer cells to evade apoptosis (6–8).

EGFR inhibitors have recently been used clinically to improve the poor prognosis of NSCLC with EGFR mutations. Almost 90% of these somatic activating mutations in EGFR consist of in-frame deletions in exon 19 and L858R point mutations in exon 21 (9,10). Gefitinib, a synthetic anilinoquinazoline, is an orally active and highly selective EGF receptor inhibitor that blocks EGF receptor autophosphorylation and subsequent signal transduction pathways implicated in the promotion of cancer cell proliferation (11). At present, gefitinib is applied to a number of human cancers and benefits some patients during treatment (12). However, while most NSCLC patients with EGFR mutations initially respond to EGFR-tyrosine kinase inhibitors (TKIs), acquired resistance ultimately develops (13).

One potential explanation for the acquired resistance may be a secondary mutation in EGFR, EGFR T790M, which occurs in ~50% of patients acquiring resistance to EGFR-TKIs (14). Additionally, MET oncogene amplification occurs in 20% of the patients with EGFR-TKI resistance (15). Amplification of MET was found to cause gefitinib resistance by driving ERBB3 (HER3)-dependent activation of PI3K, a pathway thought to be specific to the EGFR/ERBB family receptors (16). Through genetic changes, cancer cells acquire a survival advantage, such as resisting drug-induced apoptosis, to decrease the sensitivity to drug therapy. Nevertheless, this durable genetic resistance takes a relatively long time to develop, whereas other temporary or weak types of resistance mechanisms come into play earlier in treatment (17). There is evidence that the behavior of carcinomas is influenced by crosstalk between tumor cells and the host microenvironment (18,19). Stromal cells reduce the sensitivity of cancer cells to chemotherapy drugs (20,21), leading to the suggestion that co-culture of cancer cells with stromal cells may cause reduced gefitinib-induced apoptosis in cancer cells.

Since fibroblasts play a definitive role in tumor progression and drug response (22,23), in the present study we confirmed that fibroblasts efficiently induced gefitinib resistance in the HCC827 cell line which expresses EGFR exon 19 deletion mutations. In order to investigate how the susceptibility of lung cancer cells with EGFR-activating mutations to an EGFR-TKI could be affected by fibroblasts, we performed bioinformatic analysis and found that Aurora-A kinase (AURKA) overexpression played an important role in the reduced apoptosis in HCC827 cells. Further investigations showed that the p53 pathway may play a key role in the regulation of gefitinib resistance.

## Materials and methods

### Cell lines and reagents

We purchased EGFR-mutant human lung adenocarcinoma cell line HCC827 (del E746_A750) and human lung embryonic fibroblast MRC-5 cells from the Cell Bank of the Chinese Academy of Sciences. We maintained the cell lines in RPMI-1640 medium containing 10% FBS (Gibco-BRL, Gaithersburg, MD, USA) at 37°C in a humidified 5% CO_2_ atmosphere. To study the effect of cancer-associated fibroblasts on gefitinib sensitivity of HCC827 cells, one patient with histologically proven lung cancer and who underwent surgical resection in Changzheng Hospital, was enrolled. We isolated and expanded cancer-associated fibroblasts (CAFs) as previously described (24). Briefly, we distributed small pieces of tumor tissue at the bottom of 25 cm^2^ cell culture flasks that had been precoated with 2 μl of RPMI-1640 medium supplemented with 50 IU/ml penicillin, 50 μg/ml streptomycin, and 20% FBS. We incubated the tissue cultures at 37°C in humidified air with 5% CO_2_, and changed the media every 3–4 days. After 7–10 days, the cells formed homogeneous monolayers morphologically consistent with fibroblast-like cells. Immunoblots of vimentin and E-cadherin confirmed the CAF cultures (data not shown).

We purchased gefitinib from Selleck Chemicals (Houston, TX, USA) and E-cadherin, vimentin, EGFR, p-EGFR, AKT, p-Akt, ERK, p-ERK, AURKA and GAPDH antibodies were from Epitomics Inc. (Burlingame, CA, USA).

### Co-culture and viability assays

For co-culture experiments, we cultured the HCC827 cells with or without gefitinib in the lower chamber of 24-well Transwell plates (Corning Costar, Cambridge, MA, USA) and plated MRC-5 cells or CAFs in the accompanying inserts (3-μm pore size). We treated the co-cultured cells with the indicated gefitinib concentrations for 48 h. At the assay end-point, we evaluated tumor cell viability using MTT assay (Sigma-Aldrich, St. Louis, MO, USA). We completed each experiment at least 3 times, each with triplicate samples.

### Microarray analysis of gene expression

We used Affymetrix PrimeView Array chips (>60,000 probes interrogating 40,000 transcripts) to compare gene expression patterns between cDNA reserve transcripts of mono-cultured HCC827 and MRC-5 cells co-cultured with HCC827 cells under gefitinib treatment. We used the t-test to identify differences. We considered P<0.05 as a significant difference and a cut-off line of 1.1-fold change. We built gene co-expression networks based upon the KEGG database by searching interactions among genes and locating upstream and downstream genes (25).

### Transfection of the AURKA shRNA plasmid and AURKA overexpression plasmid

Dr Zhou Wang (Shanghai, China) kindly provided pGenesil and the pEGFPC3 plasmid. We cloned oligonucleotides containing the selected shRNA sequences into pGenesil. The sequences of the clones are as follows: sense, 5′-GATCCGGGCTACAGCTCCAGTTGGA TTCAAGACGTCCAACTGGAGCTGTAGCCTTTTTA-3′ and antisense, 5′-AGCTTAAAAAGGCTACAGCTCCAGTT GGACGTCTTGAATCCAACTGGAGCTGTAGCCCG-3′.

We transfected AURKA-shRNA and empty vectors into the HCC827 cells using FuGENE HD transfection agent (Promega, Madison, WI, USA) according to the manufacturer’s instructions.

We isolated total RNA from HCC827 cells and used a reverse transcription system kit (Takara, Tokyo, Japan) to synthesize the first-strand complementary DNA. We amplified the open reading frame (ORF) of AURKA using PCR with the following primer pairs: sense, 5′-ATGGACCGATCTAAAGA AAACTGCA-3′ and antisense, 5′-TCTCCCCCTGCACGATT CCTAA-3′. We further ligated the fragment containing the ORF of AURKA into the pEGFP-C3 vector, at the *Hin*dIII/*Bam*HI site and named the recombinant plasmid p-EGFPC3-AURKA. We used FuGENE HD transfecting agent (Promega) to infect the HCC827 cells with either the p-EGFPC3-AURKA or the empty vectors.

### Immunofluorescence analysis

Gefitinib-treated and untreated HCC827 cells grown on glass coverslips were fixed with 4% PBS buffered formaldehyde for 20 min, permeabilized with 0.05% Triton X-100 in PBS for 5 min, and blocked. We washed the slides followed by incubation with the rabbit anti-human AURKA antibody (1:200) for 2 h at 37°C. The slides were washed with PBS, stained with Alexa Fluor 594 (red)-conjugated matched secondary antibody and DAPI (for nuclei), and imaged with a fluorescence microscope (Olympus, Tokyo, Japan).

### Real-time quantitative reverse transcription PCR

We validated the microarray results by qPCR. We used TRIzol reagent (Invitrogen Life Technologies, Carlsbad, CA, USA) to isolate total RNA from cells. We performed RT using a reverse transcription system kit to synthesize the first-strand complementary DNA. For PCR, we used SYBR-Green dye (Takara) with a 7900HT Detection system (Applied Biosystems, Foster City, CA, USA) and normalized the results to GAPDH. We used the following primer sets: AURKA forward, 5′-GAG GCCAATGCTCAGAGAAG-3′ and reverse, 5′-AGGGAGGT TAAGGCACACCT-3; p53 forward, 5′-GGCCCACTTCACC GTACTAA-3′ and reverse, 5′-GTGGTTTCAAGGCCAGA TGT-3′; HDM2 forward, 5′-ACGACAAAGAAAACGCC ACA-3′ and reverse, 5′-ACCAGCATCAAGATCCGGAT-3′; GAPDH forward, 5′-AATTCCATGGCACCGTCA-3′ and reverse, 5′-TGGACTCCACGACGTACTCA-3′.

### Western blotting

We harvested and lysed mono- and co-cultured tumor cells treated and untreated with gefitinib for immunoblotting. We resolved equal amounts of protein samples using 8% SDS-PAGE gels and transferred the proteins to a nitrocellulose membrane. We blocked the membrane with 5% nonfat milk and 0.05% Tween-20 for 1 h at room temperature. We incubated the blots overnight at 4°C with the respective primary antibodies. Following the washes, we incubated the blots for 2 h at room temperature with the HRP-conjugated antibodies. We used ECL Plus reagent (Thermo Fisher Scientific, Waltham, MA, USA) to visualize immunoactivity. We performed densitometric analysis of the bands using Image Lab (Bio-Rad Laboratories, Inc., Hercules, CA, USA).

### Statistical analysis

We compared differences by one-way ANOVA. We used GraphPad Prism version 6.01 (GraphPad Software) for all statistical analysis. We considered P<0.05 as a statistically significant difference.

## Results

### Fibroblasts induce gefitinib resistance in lung cancer cells

**Figure 1:**
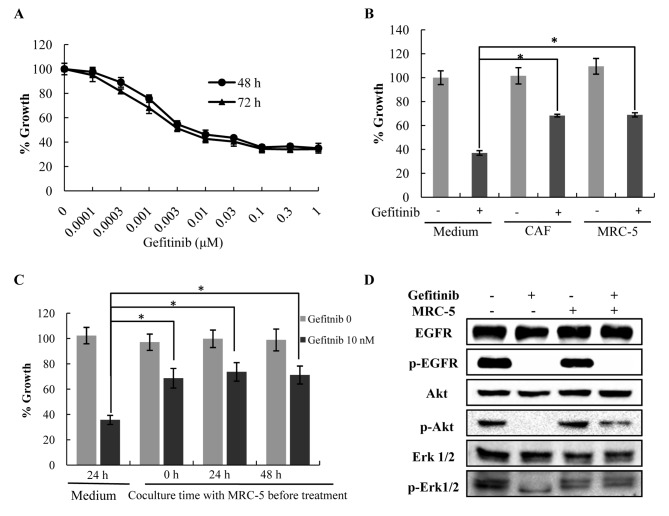
Fibroblasts induce gefitinib resistance in lung cancer cells. For A–C, the percent growth was determined by MTT assay. (A) Gefitinib at 0.3 μM resulted in a maximal decrease in the percent growth of the HCC827 cells incubated for 48 and 72 h. (B) Gefitinib at 0.3 μM decreased the percent growth of the HCC827 cells in the presence of MRC-5 cells, CAFs and medium alone. ^*^P<0.01 (one-way ANOVA). (C) Gefitinib at 0.01 μM decreased the percent growth of the HCC827 cells co-cultured with MRC-5 cells for 24 or 48 h. ^*^P<0.01 (one-way ANOVA). (D) The expression levels of p-ERK and p-Akt were increased in the HCC827 cells co-cultured with MRC-5 cells compared to the mono-culture following treatment with gefitinib, while p-EGFR expression levels did not change. EGFR, epidermal growth factor receptor; ERK, extracellular signal regulated kinase; CAFs, cancer-associated fibroblasts.

We examined the effect of co-culture with fibroblasts on the protection of HCC827 cells from gefitinib-induced apoptosis. HCC827 cells treated with 0.3 μM gefitinib resulted in a maximal 60% decrease in baseline viability at 48 and 72 h (Fig. 1A). Therefore, we used this concentration for the remainder of the experiments. Co-culture with MRC-5 cells protected HCC827 cells from gefitinib-induced apoptosis (P<0.05) (Fig. 1B). In the absence of gefitinib, MRC-5 cells did not promote the growth of HCC827 cells. Similar results were obtained using a CAF cell line. We did not find evidence of any increasing protective effect when cells were exposed for longer time periods (24 or 48 h, Fig. 1C). These results suggest that fibroblasts reduce gefitinib sensitivity in lung cancer cells with EGFR-activating mutations, regardless of the state of the fibroblasts. Moreover, we found that the expression levels of p-ERK and p-Akt increased in the HCC827 cells co-cultured with MRC-5 cells compared to the mono-culture following treatment with gefitinib, while the p-EGFR expression levels did not change. (Fig. 1D). This revealed that the MRC-5 cells prevented the reduction of p-ERK and p-Akt levels in a p-EGFR-independent manner in the HCC827 cells in the presence of gefitinib.

### Gefitinib regulates expression of AURKA in HCC827 cells

**Figure 2:**
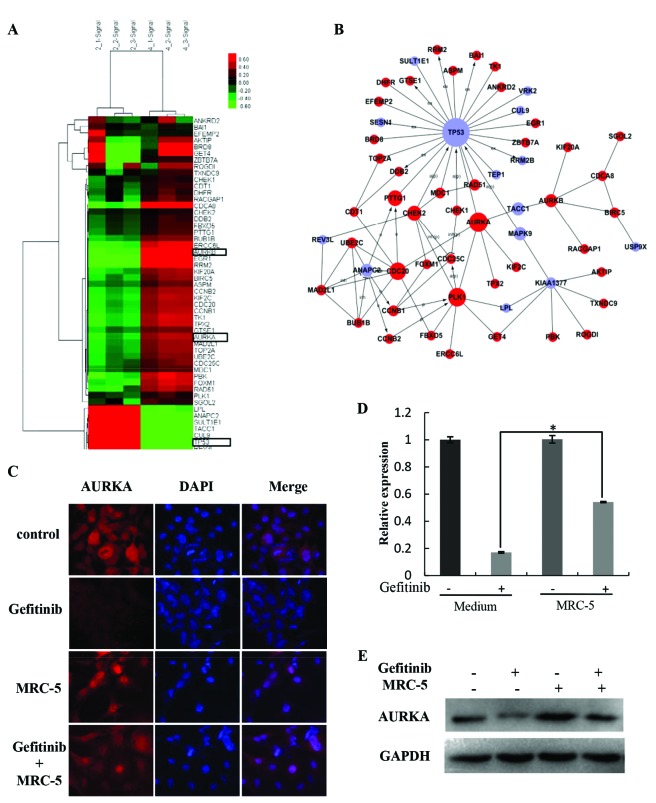
Gefitinib regulates expression of AURKA in HCC827 cells. (A) Microarray analysis demonstrating that AURKA was significantly upregulated and p53 was significantly downregulated (P<0.01) in the gefitinib-treated HCC827 cells in mono- and co-culture with MRC-5 cells. (B) Co-expression network of altered gene expression, in which AURKA and p53 are centrally located. (C) Immunocytochemistry, (D) western blot analysis and (E) PCR results showing that AURKA expression is decreased in the HCC827 cells following treatment with gefitinib to a lesser extent when co-cultured with MRC-5 cells than when in mono-culture. ^*^P<0.01 (one-way ANOVA). In C, HCC827 cells were labeled with the AURKA antibody and DAPI and imaged at magnification, ×200. AURKA, Aurora-A kinase.

In order to further explore the mechanisms underlying the different growth rates of HCC827 cells following treatment with gefitinib between mono- and co-culture with MRC-5 cells, we compared global gene expression using an Affymetrix PrimeView assay. The genes with significant change (P<0.05 and false discovery rate <0.05) were selected. Cluster analysis revealed an increased expression of AURKA and decreased expression of p53 (Fig. 2A). A co-expression network of altered gene expression was built, in which AURKA and p53 were centrally located (Fig. 2B). Since studies indicate that AURKA is involved in the regulation of resistance to various chemotherapeutic drugs, we elected to further explore the role of AURKA in reduced gefitinib sensitivity in HCC827 cells. We confirmed that in the presence of gefitinib, HCC827 cells expressed higher levels of AURKA under stromal co-cultures compared with the mono-culture cells (Fig. 2D and E). Immunofluorescence results corroborated the PCR and western blotting results (Fig. 2C). Importantly, in the absence of gefitinib, HCC827 cells co-cultured with fibroblasts expressed the same level of AURKA when compared with this level in the HCC827 cells in the mono-culture. This suggests that AURKA actively supports the survival of gefitinib-treated cells while being co-cultured with MRC-5 cells.

### Overexpression of AURKA reduces gefitinib sensitivity of HCC827 cells

**Figure 3:**
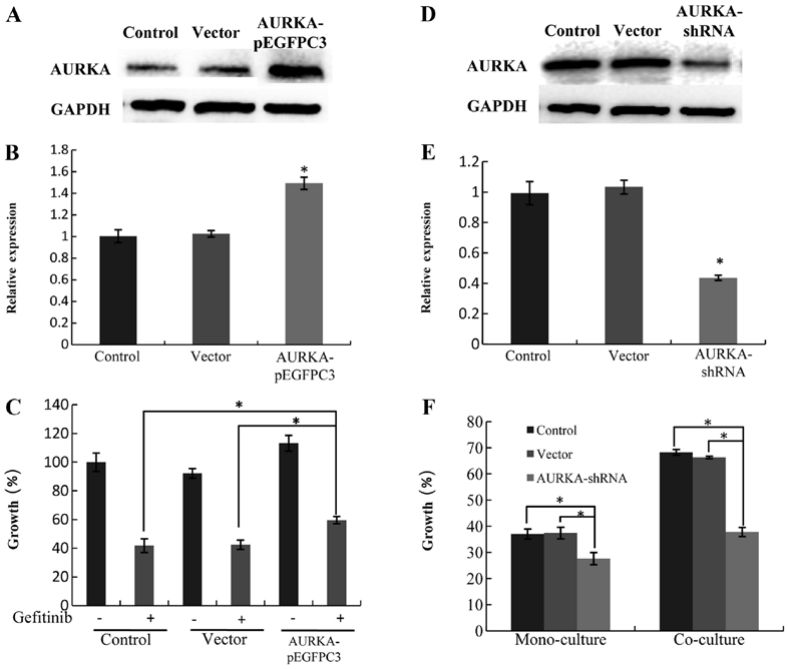
Overexpression and inhibition of AURKA influences the gefitinib sensitivity of HCC827 cells. The plasmid p-EGFPC3 containing the AURKA CDS sequence was constructed and transfected into HCC827 cells. The AURKA expression level was examined by (A) western blot analysis and (B) real-time PCR. ^*^P<0.01 (one-way ANOVA). (C) At 24 h post-transfection, HCC827 cells transfected with the p-EGFPC3 vector or overexpression plasmid were subsequently treated with gefitinib for 48 h. The percent growth was determined by MTT assay, ^*^P<0.01, (one-way ANOVA). The percent growth was determined by MTT assay, ^*^P<0.01, (one-way ANOVA). The plasmid containing the AURKA-shRNA sequence was constructed and transfected into the HCC827 cells. The AURKA expression level was examined by (D) western blot analysis and (E) real-time PCR, ^*^P<0.01 (one-way ANOVA). (F) AURKA-shRNA abrogated the protective effect of MRC-5 cells on gefitinib-induced apoptosis. The percent growth was determined by MTT assay, ^*^P<0.01, (one-way ANOVA). AURKA, Aurora-A kinase.

We constructed an overexpression plasmid of AURKA to investigate whether AURKA overexpression in HCC827 cells affects the protective effect caused by MRC-5 cells against gefitinib-induced apoptosis (Fig. 3A and B). We found that AURKA overexpression reduced the gefitinib sensitivity of HCC827 cells (Fig. 3C), suggesting that AURKA upregulation in HCC827 cells may be the leading cause of resistance to gefitinib when co-cultured with fibroblasts.

### Inhibition of AURKA restores gefitinib sensitivity in HCC827 cells co-cultured with MRC-5 cells

We constructed an AURKA-shRNA plasmid (Fig. 3D and E) to investigate whether AURKA knockdown influences the gefitinib sensitivity of HCC827 cells. We found that AURKA-shRNA abrogated the protective effect of MRC-5 cells against gefitinib-induced apoptosis (Fig. 3F).

### AURKA influences gefitinib sensitivity by regulating p53 signaling

**Figure 4:**
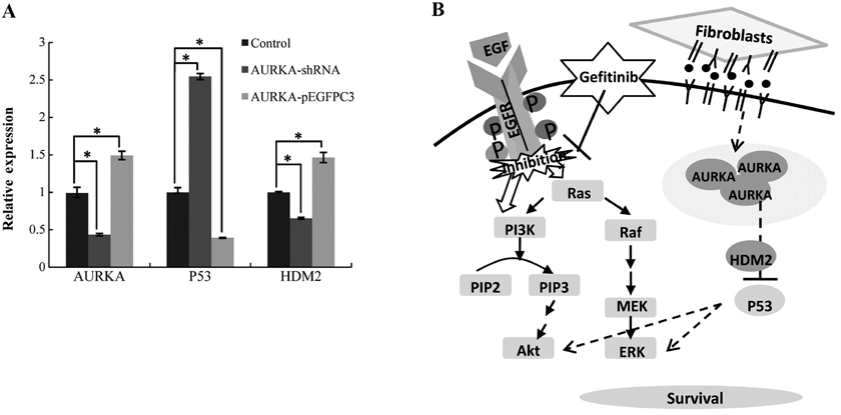
AURKA influences the gefitinib sensitivity by regulating p53 signaling. (A), inhibition of AURKA upregulated expression of p53 and downregulated expression of HDM2. Conversely, overexpression of AURKA downregulated p53 expression and upregulated HDM2 expression. ^*^P<0.01 (one-way ANOVA). (B) A model illustrating the mechanism of fibroblast-induced gefitinib resistance. Co-culture with fibroblasts upregulated the AURKA expression level in HCC827 cells. It has been reported that AURKA promotes tumor growth and cell survival through regulation of HDM2-induced ubiquitination and inhibition of p53. We proposed that AURKA upregulation inhibited p53 expression through HDM2 and therefore induced activated Akt and ERK to promote the survival of HCC827 cells.

We investigated whether decreased expression of AURKA affects the p53 signaling pathway. Inhibition of AURKA upregulated expression of p53 and downregulated expression of HDM2 (Fig. 4A). Conversely, overexpression of AURKA downregulated p53 expression and upregulated HDM2 expression. It has been reported that AURKA promotes tumor growth and cell survival through regulation of HDM2-induced ubiquitination and inhibition of p53 (26). From the above results, we propose that fibroblasts induce gefitinib resistance by regulating the expression of AURKA, thus inhibiting the p53 signaling pathway (Fig. 4B).

## Discussion

In the present study, we confirmed that co-culture with fibroblasts partially reduced the sensitivity of HCC827 cells to gefitinib-induced apoptosis. Since NSCLC EGFR-TKI therapy blocks the activation of EGFR, we evaluated expression levels of p-EGFR, a molecular correlate of EGFR activity, and its downstream effectors, p-ERK and p-Akt, in HCC827 cells in mono- and co-culture with MRC-5 cells. Co-culture with MRC-5 cells did not protect HCC827 cells from gefitinib induced pEGFR inhibition but significantly increased p-Akt and p-ERK activation compared with the mono-culture cells. Without gefitinib, the activation of EGFR and its downstream effectors in HCC827 cells did not change when co-cultured with MRC-5 cells. Our data, along with previous research, demonstrating that activation of MAPK pathways promote survival of cancer cells (27) suggest that co-culture with fibroblasts partially abrogates the gefitinib-induced inhibitory effect of downstream molecules in an EGFR-independent manner in HCC827 cells.

The tumor microenvironment is important for tumor progression. Fibroblasts have been linked to several activities that promote tumor progression, including angiogenesis, progressive genetic instability, deregulation of antitumor immune responses, enhanced metastasis, and enhanced growth (28,29). Several prospective studies indicate that HGF secreted by fibroblasts reduces gefitinib sensitivity in tumor cells (30), but the underlying mechanism by which cancer cells survive, remains poorly understood.

Through bioinformatics analysis we found that following treatment with gefitinib, AURKA expression was higher in the HCC827 cells co-cultured with fibroblasts compared with the mono-cultured cells. AURKA is a member of the Aurora kinase family of serine/threonine kinases whose structures and functions are highly conserved in different model organisms (31). They play an important role during cell mitosis, such as promoting the cell to enter mitosis, assisting in centrosome maturation and separation, assembling and maintaining spindles, segregating chromosomes, and aiding in cytokinesis (32,33). Previous studies showed that AURKA phosphorylates p53 at Ser315, leading to its ubiquitination by Mdm2 and proteolysis. Knockdown of AURKA results in less phosphorylation of p53 at Ser315, greater stability of p53, and cycle arrest at G2-M (33). Overexpression of Aurora kinase has been observed in some tumor cells and aberration in Aurora kinases has proven to be involved in tumorigenesis (34). It has been reported that AURKA promotes tumor growth and cell survival through regulation of HDM2-induced ubiquitination and inhibition of p53 (35). Consistent with this, we indentified that the expression levels of p53 and HDM2 are regulated by AURKA in HCC827 cells. Notably, the expression level of AURKA in co-cultured HCC827 cells did not change compared with the mono-cultured HCC827 cells in the absence of gefitinib, suggesting that fibroblasts promoted tumor cell survival only in the presence of gefitinib. We also found that AURKA inhibition alone led to reduced survival in the HCC827 cells. AURKA plays an important role in mediating gefitinib resistance as shown by the restoration of gefitinib resistance of HCC827 cells co-cultured with fibroblasts in the presence of AURKA-shRNA.

Taken together, our results provide genetic evidence that the tumor microenvironment is a novel modulator of resistance to gefitinib. Importantly, increased cell survival did not result from reduced p-EGFR inhibition, but from downstream EGFR effectors. Moreover, we also demonstrated that decreased drug sensitivity was caused by increased expression of AURKA, which may rescue cells from gefitinib-induced apoptosis by p53 signaling pathway inhibition. In conclusion, our findings have important implications in cancer-targeted therapy. AURKA inhibition enhances the efficacy of EGFR-TKIs and reverses acquired resistance resulting from fibroblasts in the microenvironment. Consequently, preventing AURKA upregulation may provide valuable enhancement of the efficacy of specific targeted therapy regimens.

## References

[1] Ferlay J, Shin HR, Bray F, Forman D, Mathers C, Parkin DM (2010). Estimates of worldwide burden of cancer in 2008: GLOBOCAN 2008. Int J Cancer.

[2] Xue C, Hu Z, Jiang W (2012). National survey of the medical treatment status for non-small cell lung cancer (NSCLC) in China. Lung Cancer.

[3] Yarden Y (2001). The EGFR family and its ligands in human cancer. Signaling mechanisms and therapeutic opportunities. Eur J Cancer.

[4] Schlessinger J (2000). Cell signaling by receptor tyrosine kinases. Cell.

[5] Zhu H, Cao X, Ali-Osman F, Keir S, Lo HW (2010). EGFR and EGFRvIII interact with PUMA to inhibit mitochondrial translocalization of PUMA and PUMA-mediated apoptosis independent of EGFR kinase activity. Cancer Lett.

[6] Leu CM, Chang C, Hu C (2000). Epidermal growth factor (EGF) suppresses staurosporine-induced apoptosis by inducing mcl-1 via the mitogen-activated protein kinase pathway. Oncogene.

[7] Huang SH, Li Y, Chen HG, Rong J, Ye S (2013). Activation of proteinase-activated receptor 2 prevents apoptosis of lung cancer cells. Cancer Invest.

[8] Wang Q, Greene MI (2005). EGFR enhances survivin expression through the phosphoinositide3 (PI-3) kinase signaling pathway. Exp Mol Pathol.

[9] Lynch TJ, Bell DW, Sordella R (2004). Activating mutations in the epidermal growth factor receptor underlying responsiveness of non-small-cell lung cancer to gefitinib. N Engl J Med.

[10] Paez JG, Jänne PA, Lee JC (2004). EGFR mutations in lung cancer: correlation with clinical response to gefitinib therapy. Science.

[11] Woodburn JR (1999). The epidermal growth factor receptor and its inhibition in cancer therapy. Pharmacol Ther.

[12] Wakeling AE, Guy SP, Woodburn JR (2002). ZD1839 (Iressa): an orally active inhibitor of epidermal growth factor signaling with potential for cancer therapy. Cancer Res.

[13] Kobayashi S, Boggon TJ, Dayaram T (2005). EGFR mutation and resistance of non-small-cell lung cancer to gefitinib. N Engl J Med.

[14] Balak MN, Gong Y, Riely GJ (2006). Novel D761Y and common secondary T790M mutations in epidermal growth factor receptor-mutant lung adenocarcinomas with acquired resistance to kinase inhibitors. Clin Cancer Res.

[15] Bean J, Brennan C, Shih JY (2007). MET amplification occurs with or without T790M mutations in EGFR mutant lung tumors with acquired resistance to gefitinib or erlotinib. Proc Natl Acad Sci USA.

[16] Engelman JA, Zejnullahu K, Mitsudomi T (2007). MET amplification leads to gefitinib resistance in lung cancer by activating ERBB3 signaling. Science.

[17] Baguley BC (2010). Multiple drug resistance mechanisms in cancer. Mol Biotechnol.

[18] Liotta LA, Kohn EC (2001). The microenvironment of the tumor-host interface. Nature.

[19] Witz IP (2008). Tumor-microenvironment interactions: dangerous liaisons. Adv Cancer Res.

[20] Gottesman MM (2002). Mechanisms of cancer drug resistance. Annu Rev Med.

[21] Cukierman E, Bassi DE (2012). The mesenchymal tumor microenvironment: a drug-resistant niche. Cell Adh Migr.

[22] Flach EH, Rebecca VW, Herlyn M, Smalley KS, Anderson AR (2011). Fibroblasts contribute to melanoma tumor growth and drug resistance. Mol Pharm.

[23] Ostman A, Augsten M (2009). Cancer-associated fibroblasts and tumor growth - bystanders turning into key players. Curr Opin Genet Dev.

[24] Johansson AC, Ansell A, Jerhammar F (2012). Cancer-associated fibroblasts induce matrix metalloproteinase-mediated cetuximab resistance in head and neck squamous cell carcinoma cells. Mol Cancer Res.

[25] Jansen R, Greenbaum D, Gerstein M (2002). Relating whole-genome expression data with protein-protein interactions. Genome Res.

[26] Sehdev V, Katsha A, Arras J (2014). HDM2 regulation by AURKA promotes cell survival in gastric cancer. Clin Cancer Res.

[27] Zimmer S, Kahl P, Buhl TM (2009). Epidermal growth factor receptor mutations in non-small cell lung cancer influence downstream Akt, MAPK and Stat3 signaling. J Cancer Res Clin Oncol.

[28] Ostman A, Augsten M (2009). Cancer-associated fibroblasts and tumor growth - bystanders turning into key players. Curr Opin Genet Dev.

[29] Bhowmick NA, Neilson EG, Moses HL (2004). Stromal fibroblasts in cancer initiation and progression. Nature.

[30] Donev IS, Wang W, Yamada T (2011). Transient PI3K inhibition induces apoptosis and overcomes HGF-mediated resistance to EGFR-TKIs in EGFR mutant lung cancer. Clin Cancer Res.

[31] Giet R, Prigent C (1999). Aurora/Ipl1p-related kinases, a new oncogenic family of mitotic serine-threonine kinases. J Cell Sci.

[32] Gautschi O, Heighway J, Mack PC, Purnell PR, Lara PN, Gandara DR (2008). Aurora kinases as anticancer drug targets. Clin Cancer Res.

[33] Nishimura Y, Endo T, Kano K, Naito K (2009). Porcine Aurora A accelerates Cyclin B and Mos synthesis and promotes meiotic resumption of porcine oocytes. Anim Reprod Sci.

[34] Anand S, Penrhyn-Lowe S, Venkitaraman AR (2003). Aurora-A amplification overrides the mitotic spindle assembly checkpoint, inducing resistance to taxol. Cancer Cell.

[35] Katayama H, Sasai K, Kawai H (2004). Phosphorylation by aurora kinase A induces Mdm2-mediated destabilization and inhibition of p53. Nat Genet.

